# Young age and the risk of violent behaviour in people with severe mental disorders: prospective, multicentre study

**DOI:** 10.1192/bjo.2021.1047

**Published:** 2021-12-01

**Authors:** Rocco Micciolo, Giorgio Bianconi, Luisa Canal, Massimo Clerici, Maria Teresa Ferla, Camilla Giugni, Laura Iozzino, Giulio Sbravati, Giovanni Battista Tura, Antonio Vita, Laura Zagarese, Giovanni de Girolamo, for the Violence Risk and Mental Disorder 2 (VIORMED-2) Group

**Affiliations:** Department of Psychology and Cognitive Sciences, University of Trento, Italy; Department of Mental Health, ASST Ovest Milanese, Milano, Italy; Department of Psychology and Cognitive Sciences, University of Trento, Italy; School of Medicine and Surgery, University of Milan-Bicocca, Monza, Italy; and Department of Psychiatry, ASST Monza, Italy; Department of Mental Health, ASST Rhodense G.Salvini di Garbagnate, Milano, Italy; Unit of Epidemiological and Evaluation Psychiatry, IRCCS Istituto Centro San Giovanni di Dio Fatebenefratelli, Brescia, Italy; Unit of Epidemiological and Evaluation Psychiatry, IRCCS Istituto Centro San Giovanni di Dio Fatebenefratelli, Brescia, Italy; Unit of Epidemiological and Evaluation Psychiatry, IRCCS Istituto Centro San Giovanni di Dio Fatebenefratelli, Brescia, Italy; Clinical Psychiatry Department, IRCCS Istituto Centro San Giovanni di Dio Fatebenefratelli, Brescia, Italy; Department of Mental Health, ASST Spedali Civili of Brescia, Italy; Unit of Epidemiological and Evaluation Psychiatry, IRCCS Istituto Centro San Giovanni di Dio Fatebenefratelli, Brescia, Italy; Unit of Epidemiological and Evaluation Psychiatry, IRCCS Istituto Centro San Giovanni di Dio Fatebenefratelli, Brescia, Italy

**Keywords:** Mental disorders, young age, violence, risk prediction, impulsivity

## Abstract

**Background:**

During adolescence and young adulthood people appear to be more prone to violent behaviour. A greater tendency to violent behaviour appears to be associated with hyperactivity, impulsivity and low tolerance for frustration and provocation in social settings.

**Aims:**

This prospective cohort study aimed to evaluate rates of violent behaviour among young people with mental disorders, compared with older age groups.

**Method:**

A total of 340 individuals with severe mental disorders (125 living in residential facilities and 215 out-patients) were evaluated at baseline with the SCID-I and II, Brief Psychiatric Rating Scale, Specific Level of Functioning scale, Brown–Goodwin Lifetime History of Aggression scale, Buss–Durkee Hostility Inventory, Barratt Impulsiveness Scale and State–Trait Anger Expression Inventory-2. Aggressive behaviour was rated every 15 days with the Modified Overt Aggression Scale (MOAS).

**Results:**

The sample comprised 28 individuals aged 18–29 years, 202 aged 30–49 and 110 aged 50 and over. Younger age was associated with a personality disorder diagnosis, substance use disorder, being single and employed. These results were confirmed even controlling for the gender effect. The patterns of the cumulative MOAS mean scores showed that younger (18–29 years old) individuals were significantly more aggressive than older (≥50) ones (*P* < 0.001).

**Conclusions:**

This study highlights how young age in people with severe mental disorders is correlated with higher levels of impulsivity, anger and hostility, confirming previous analyses. Our results may assist clinicians in implementing early interventions to improve anger and impulsivity control to reduce the risk of future aggressive behaviours.

A large proportion of violent, aggressive and antisocial behaviours emerges during adolescence and young adulthood.^1–5^. Young males in particular seem to be more inclined to violent behaviour;^4–6^ among them, hyperactivity, impulsivity, low tolerance for frustration and social provocations, and a risk-taking tendency seem to be associated with a greater tendency to violent behaviour.^2,6^ As for the gender differences related to violent behaviour, males traditionally show higher rates of aggression than females, especially in terms of physical violence, whereas females exhibit a more indirect type of aggression.^7,8^ Response disinhibition, impulsivity and risk-taking are also usually associated with substance use disorders (SUDs) and antisocial personality disorder.^9–12^ Among these psychological variables, impulsivity may play a special role: it is the tendency to exhibit rapid, unplanned behaviour in response to a stimulus without assessing the long-term consequences, and it aims for immediate reward.^10,11,13^ Impulsivity has its peak in adolescence and generally decreases with advancing age, owing to the development of cognitive control skills.^10,14,15^ SUDs, and in particular alcohol use disorder, are also linked to an increased risk of aggression,^16^ often jointly with the presence of antisocial traits and impulsivity.^17,18^ There is a bidirectional relationship between high levels of impulsivity, externalising behaviour and SUDs.^19–22^ Indeed, impulsivity and emotional regulation are closely associated with SUDs, although SUDs may sometimes be a coping strategy for the stress caused by adverse events.^22–24^ Hence, impulsivity and emotional dysregulation might be triggering factors, but also consequences of SUDs, predicting possible relapse in individuals with this condition.^22^

## Aims

The aim of this paper is to prospectively assess the risk of aggressive and violent behaviour among individuals with mental disorders in young (18–29 years old) compared with older age groups. We hypothesise that younger individuals will exhibit higher rates of aggressive and violent behaviour also controlling for a number of variables, including SUDs, impulsivity and externalising behaviour.

## Method

### Design overview and participants

Violence Risk and Mental Disorders (VIORMED) is a prospective cohort study with a baseline cross-sectional comparative design, followed by a 1-year follow-up observation period. This study included patients living in residential facilities and out-patients under the care of four Departments of Mental Health in northern Italy. Many details about both the study settings and the design can be found in previous publications.^25,26^ Inclusion criteria were a primary psychiatric diagnosis and age between 18 and 65 years. Exclusion criteria included a diagnosis of organic mental disorder, intellectual disability, dementia or sensory deficits. The selection of these patients was based only on a comprehensive and detailed documentation (as reported in clinical records) of a history of severe violent behaviour(s).^26^

Written informed consent was obtained from all patients. Ethical approval was granted by the ethical committee of the coordinating centre (IRCCS Saint John of God, Fatebenefratelli, no. 64/2014) and by the ethical committees of all the recruiting centres.

### Measures and assessments

Sociodemographic characteristics, clinical and treatment-related data and information about their history of violence were collected for all participants recruited. The Structured Clinical Interview for DSM-IV Axis I^27^ and Axis II^28^ (SCID-I and SCID-II) were administered to confirm clinical diagnoses. Symptom severity, personal and social functioning were assessed using the Brief Psychiatric Rating Scale–Expanded (BPRS-E)^29^ and the Specific Level of Functioning scale.^30^

Aggression and violence, impulsivity and hostility were evaluated using the Brown–Goodwin Lifetime History of Aggression (BGLHA),^31^ the Buss–Durkee Hostility Inventory (BDHI)^32^ and the Barratt Impulsiveness Scale Version 11 (BIS-11).^33^ Anger was measured using the State–Trait Anger Expression Inventory-2 (STAXI-2).^34^ Details about these tools can be found in Barlati et al;^26^ all these tools have been validated in Italy.

### Monitoring of aggressive and violent behaviour

The treating clinician, or a close family member for some out-patients, rated each participant on the Modified Overt Aggression Scale (MOAS)^35^ every 2 weeks during the 1-year follow-up, giving a total of 24 MOAS evaluations for each individual. The MOAS includes four aggression subdomains: verbal, against objects, against self and interpersonal physical. In each evaluation the score ranges from 0 (no aggression) to 40 (maximum grade of aggression), so that the individual MOAS total weighted score for the 1-year period could range from 0 to 960. We will refer to the weighted MOAS total score (our primary outcome) simply as the MOAS score.

### Statistical analyses

The analysis of aggressive and violent behaviour was conducted by evaluating the MOAS scores in all 24 assessments, and their trends were estimated by calculating cumulative means, modifying the technique outlined in Lawless & Nadeau^36^ and in Canal & Micciolo.^37^

This approach, which uses the cumulative mean of all MOAS scores, produces a graphical display of the participants’ patterns of behaviour. In this framework, the estimation of cumulative means is rather simple. Going into detail, if *k* is the number of participants constant over time, *t* is the evaluation time (*t* = 1, 2, …, 24) and *S_t_* is the total MOAS score observed over the interval [1, *t*] evaluated by adding up all the individual MOAS scores observed from the first up to the *t*-th evaluation, the cumulative mean function of the MOAS score at evaluation *t* is calculated as *M_t_* = *S_t_/k*. If *M_t_* is the arithmetic mean of the MOAS scores at time *t*, then *M*_1_ = *m*_1_, *M*_2_ = *m*_1_ + *m*_2_ and *M*_t_ = *m*_1_ + *m*_2_ + …*m_t_* are the sum of the means of the MOAS scores observed up to time *t*. For instance, if the means of the MOAS scores observed at times 1, 2, 3 and 4 are 1.41, 0.95, 1.06 and 0.97, the cumulative means of the MOAS scores are 1.41, 2.36, 3.42 and 4.39 respectively.

To measure the pattern of aggression, the area under the corresponding curves (AUC) has been computed using a trapezoidal rule. It is interesting to note that, by the properties of the arithmetic mean, the mean of the AUCs for the 24 cumulative MOAS scores for each participant corresponds to the AUC for the cumulative means of the MOAS scores.

To compare categorical data, the χ^2^-test or Fisher's exact test was used as appropriate. For quantitative data, an analysis of variance (ANOVA) or Student's *t*-test was employed. The assumption of normality was investigated by a visual inspection of the distribution of variables using quantile–quantile (Q–Q) plots. The association between age and other quantitative variables was quantified using Pearson's correlation coefficient.

We used four different statistical techniques to explain the relationship between age and the pattern of aggression quantified using the AUC for the MOAS scores, all of which allow for a non-linear association: smoothing splines, local regression, super smoother and kernel smoother; an in-depth description of these techniques can be found in Venables & Ripley.^38^

Finally, logistic regression was employed to quantify the prognostic role of age, adjusting for other selected variables, in predicting the probability of having one or more episodes of aggression.

The likelihood ratio test was used, at a level of significance of 5%, to assess whether age was a significant predictor of episodes of aggression, after adjusting for the effect of other variables; the 95% confidence intervals for the adjusted odds ratios were also calculated. All statistical analyses were carried out using R 3.6.2 for Windows (R Foundation for Statistical Computing)^39^ and the MASS package (version 7.3-51.4).^38^

## Results

### Sample characteristics

We recruited 340 participants: 181 (53.2%) had a history of violence, whereas the remaining 159 (46.8%) were not know to have behaved violently during their lifetime. Of these 340 individuals, 177 (52.1%) had a diagnosis of schizophrenia, 90 (26.5%) met criteria for a personality disorder and 73 (21.4%) had other mental disorders (bipolar disorder, 31; unipolar depression, 18; and severe anxiety disorder, 14). Of the total, 125 individuals (36.8%) were living in residential facilities and 215 (63.2%) were out-patients. Most (81.5%) were males. Table 1 shows sociodemographic and clinical characteristics stratified by gender. Significant gender differences were found for civil status, diagnosis, treatment setting and history of violence.

**Table 1:** Gender differences according to different sociodemographic and clinical characteristics

	Males	Females	χ^2^	*P*^a^
Age, years	277	63	2.48	0.290
<30	6.1	11.1		
30−49	57.0	49.2		
≥50	36.8	39.7		
Civil status	277	63	7.31	**0.006**
With partner	14.8	30.2		
Single	85.2	69.8		
Education	277	63	0.56	0.372
Low level	69.3	63.5		
Medium-high level	30.7	36.5		
Occupation	275	61	1.02	0.292
Employed	31.6	39.3		
Unemployed	68.4	60.7		
Diagnosis	277	63	15.20	**<0.001**
Schizophrenia	55.2	38.1		
Personality disorder	22.0	46.0		
Any other	22.7	15.9		
Treatment setting	276	63	5.01	**0.020**
Out-patient	39.9	23.8		
Residential	60.1	76.2		
Previous history of violence	277	63	3.88	**0.037**
Yes	56.0	41.3		
No	44.0	58.7		
Current SUD	258	61	0.07	0.824
No	88.0	90.2		
Yes	12.0	9.8		

SUD, substance use disorder.

a. Bold denotes significance at *P <* 0.05.

A significant difference in mean age was found for a number of variables for the entire sample: as regards diagnosis, participants with personality disorders were younger (mean 42.5 years, s.d. = 10.7 *v.* mean 46.3, s.d. = 10.2); single participants were younger (mean 44.7 years, s.d. = 10.4 *v.* mean 47.7, s.d. = 9.7); employed participants were younger (mean 43.5 years, s.d. = 8.5 *v.* mean 46.1, s.d. = 11); and participants with a history of SUD were younger (mean 41.8 years, s.d. = 9.7 *v.* mean 46.1, s.d. = 10.5). As regards medication prescription patterns, there was no difference in mean age (additional data are given in Online Resource 1, available at https://doi.org/10.1192/bjo.2021.1047).

The percentage of single and unemployed participants was not significantly different between participants with and without history of violence. Participants with a history of violence had a significantly lower level of education, with only 26.0% achieving a medium-high educational level compared with 38.4% of participants without a violence history (*P* = 0.019); the educational level was also significantly different among the diagnostic groups (*P* = 0.027): 25.4% of participants with schizophrenia, 36.7% of those with personality disorders and 41.1% of those with other diagnoses achieved a medium-high educational level. As far as civil status is concerned, a highly significant difference (*P* < 0.001) was found when diagnostic groups were considered: 92.7% of participants with schizophrenia, 78.4% of those with personality disorders and 79.8% of those with other diagnoses were single.

Table 2 shows the correlation coefficients between age and scores on the selected rating scales. In general, they were low in absolute value (under |0.23|), but some of them showed a significant, if weak, association with age.

**Table 2:** Correlation coefficients between age and scores of selected rating scales

Scale	*r*	*P*
BPRS-E		
Affect (anxiety)	0.154	0.006
Activation	0.051	0.358
Negative symptoms	0.191	0.001
Positive symptoms	0.176	0.001
Total	0.182	0.001
BGLHA	−0.170	0.010
BIS–11	−0.077	0.243
BDHI	−0.066	0.317
STAXI-2 state anger at *T*_0_	0.062	0.271
STAXI-2 trait anger at *T*_0_	−0.009	0.878
STAXI-2 anger expression index at *T*_0_	−0.001	0.991
SLOF		
Physical functioning	−0.226	<0.001
Self-care	−0.225	<0.001
Interpersonal relationships	−0.195	<0.001
Social acceptability/adjustment	0.037	0.502
Activities	−0.127	0.022
Work skills	−0.195	<0.001

BPRS-E, Brief Psychiatric Rating Scale – Expanded; BGLHA, Brown–Goodwin Lifetime History of Aggression scale; BIS-11, Barratt Impulsiveness Scale Version 11; BDHI, Buss–Durkee Hostility Inventory; STAXI-2, State–Trait Anger Expression Inventory-2; SLOF, Specific Level of Functioning scale.

### Age and MOAS scores

Fifteen participants (11 with a history of violence and 4 without) had more than two missing MOAS evaluations and so were not considered in these analyses. Participants with up to two missing MOAS evaluations were computed by the moving average estimation method.

**Fig. 1:**
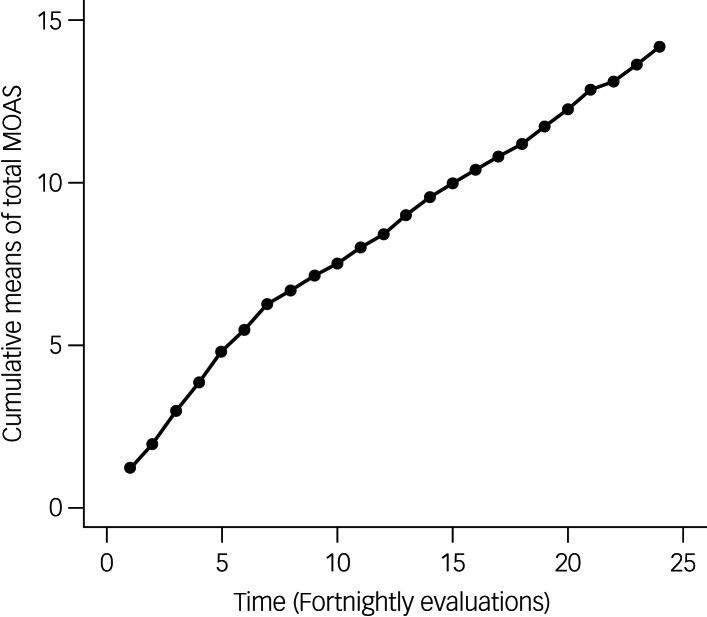
Trend in cumulative means of total scores on the Modified Overt Aggression Scale (MOAS) over time.

The cumulative MOAS mean scores (cMOAS) for the 24 fortnightly evaluations increased over time, with a clear two-phase linear trend (Fig. 1).

More specifically, a linear increase was observed between the first and the seventh MOAS evaluations; the correlation between the evaluation time and the first seven cMOAS mean scores was 0.9988: therefore, in this time span, MOAS means remained approximately constant (around the value of 0.855). After the seventh and up to the last evaluation, the observed pattern of cMOAS was again linear (with a correlation of 0.9995) but with a lower slope (i.e. a lower aggression); that is, from MOAS points 7 to 24, MOAS means remained approximately constant (around the value of 0.467).

For 121 participants the AUC = 0 (i.e. all MOAS scores were equal to 0): 104 were males (37.5%) and 17 were females (27.0%); for the remaining 219 participants the AUC > 0, and of these 173 were males (62.5%) and 46 females (73.0%). Fisher's exact test yielded a *P*-value of 0.145, showing that these percentages were not significantly different between males and females.

Among participants with an AUC > 0, the mean age was 43.4 years for males and 43.6 years for females (s.d. = 10.4 and s.d. = 11.3 years respectively). Among participants with an AUC = 0, the mean age was 47.9 years for males and 52.6 years for females (s.d. = 8.9 and s.d. = 9.0 years respectively). An ANOVA showed that mean age was not significantly different between males and females (*F* = 1.12; *P* = 0.29), whereas the difference in mean age between participants with an AUC = 0 and those with an AUC > 0 was highly significantly (*F* = 20.4; *P* < 0.001).

To evaluate the longitudinal pattern of violent behaviour (employing the cumulative means of total MOAS scores) according to age, three age groups were defined: 18–29 (*n* = 28), 30–49 (*n* = 202) and ≥50 years (*n* = 110). The cut-off thresholds were found employing four different smoothing techniques to evaluate the relationship between age and the AUC for the cumulative MOAS scores for each participant (see supplementary Fig. 1).

**Fig. 2:**
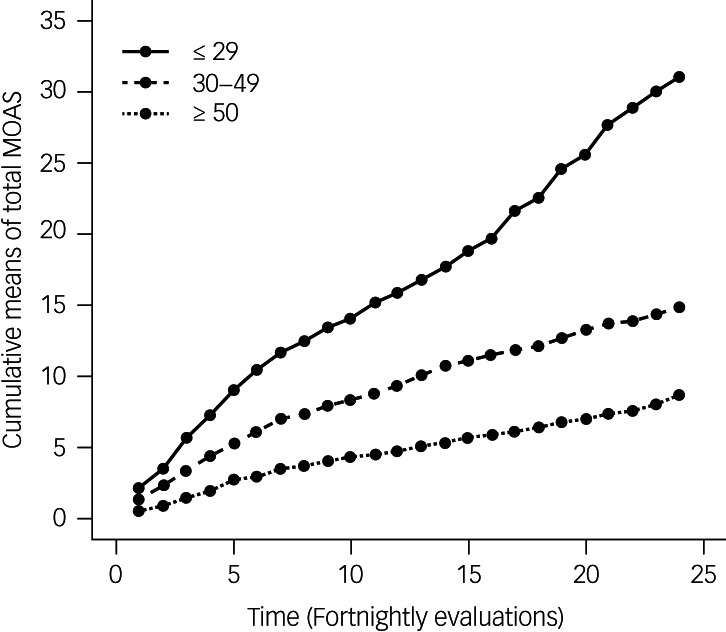
Cumulative means of total scores on the Modified Overt Aggression Scale (MOAS) in three age groups over time.

Figure 2 shows the pattern of the cMOAS scores according to age category. Younger patients showed more overt aggression than older patients. The AUCs for the cMOAS scores of the three age groups were respectively 389, 214 and 111. It is interesting to note that the ratio between the AUCs for the first and second age groups (1.82) was quite similar to the ratio for the second and third age groups (1.92), indicating a sort of ‘linear trend’ in aggressive and violent behaviour associated with age. Among males the AUCs for the cMOAS scores for the three age groups were respectively 389, 214 and 111, and among females they were 401, 296, 34. Supplementary Fig. 2 shows the cMOAS pattern stratified by age groups separately for males and females.

A cMOAS AUC = 0 was seen for only 2 out of the 28 participants between 18 and 29 years of age (7.1%) (they were males), about one-third (32.2%) of the 202 participants between 30 and 49 years of age (60 males and 5 females) and about half (49.1%) of the 110 participants over 49 years (42 males and 12 females). These three percentages were highly significantly different (χ^2^ = 19.7; *P* < 0.001), with a highly significant linear trend (*P* < 0.001). On the other hand, no difference was found when comparing the mean values of logarithmically transformed positive AUCs for the age groups (*P* = 0.224), even taking the effect of gender into account.

Generally, the pattern of cMOAS scores shown in Fig. 2 was replicated when the analysis was repeated within categories of selected variables (e.g. diagnosis, group, setting, SUD, medications); these patterns are shown in supplementary Figs 3–5. Numerically, older (≥50) participants always showed the lowest MOAS scores; the cMOAS pattern for younger participants (18–29) generally indicated the highest aggression.

### Aggressive and violent behaviour and moderating variables

To evaluate whether the probability of not showing any aggressive or violent behaviour at all remained associated with age groups after having taken into account the effect of selected variables (e.g. diagnosis, group, setting, SUDs, medications), a logistic regression analysis was employed. The results are shown in Table 3.

**Table 3:** Odds ratios and 95% confidence intervals of various models fitted with logistic regression^a^

Variable	≥50 years *v.* 30–49 years			≥50 years *v.* 18–29 years			LRT	*P*
	OR	95% CI		OR	95% CI			
		Lower	Upper		Lower	Upper		
Unadjusted (raw)	6.17	1.77	39.01	12.54	3.51	80.19	22.0	<0.001
Adjusted for
Gender	5.97	1.71	37.80	12.50	3.49	80.10	22.2	<0.001
Diagnosis	6.04	1.68	38.80	10.95	2.96	71.13	17.0	<0.001
Group	6.44	1.82	41.01	13.42	3.70	86.52	22.6	<0.001
Treatment setting	5.95	1.70	37.72	12.34	3.44	79.14	21.8	<0.001
Current SUD	6.40	1.82	40.65	11.82	3.28	75.89	19.4	<0.001
Medication group	5.77	1.64	36.59	11.85	3.30	76.02	21.0	<0.001
All previous variables	6.95	1.84	45.87	12.83	3.30	85.86	17.5	<0.001

LRT, likelihood-ratio test; SUD, substance use disorder.

a. The dependent variable is the probability of having a cumulative Modified Overt Aggression Scale score equal to zero. Raw refers to the model including only age as a categorical independent variable. The other rows show the ORs (together with associated 95% CIs) for the age category, adjusted for the variable indicated in column 1. The last row refers to the logistic model including age and the other six considered variables.

Older participants showed the highest probability of displaying no aggressive or violent behaviour at all, and younger participants showed the lowest, even after taking into account the joint effect of these selected variables. Both raw and adjusted odds ratios were quite similar and significantly different from 1 (the *P*-values of the likelihood-ratio test for the age effect, both raw and adjusted, were always highly significant and well below 0.001; Table 3). The odds of displaying no aggressive behaviour at all for participants ≥50 years old were about six times those for participants aged 30–49 and were about twelve times those for younger participants.

## Discussion

It has been long known that violent behaviour is associated with a number of static risk factors, such as male gender and young age.^1,2,4–6^ Recent comprehensive reviews on the topic have confirmed the higher risk of aggressive and violent behaviour among males.^40^ Dynamic factors, such as impulsivity, risk-taking behaviour and SUD, also correlate with a higher risk of violent behaviour.^2,10,12,40–42^ This study includes, to our knowledge, the longest follow-up of aggressive and violent behaviour, with participants monitored every 2 weeks with the MOAS.^43^

In this study we found that during the 1-year follow-up of participants with severe mental disorders, those aged 18–29 years had a risk of violent behaviour 12 times higher than those aged 50–65 years old, and the odds ratio remained high, even controlling for other variables (e.g. gender, diagnosis, group, setting, SUD, medications).

Sociodemographic characteristics, such as being single and employed, and clinical features, such as a recent history of SUD, were also associated with young age.

### Clinical correlates of aggressive and violent behaviour

In our sample of psychiatric patients, a high proportion of younger patients met diagnostic criteria for personality disorders as assessed using the SCID-II.^27,28^ Impulsivity and risk-taking are prominent features of specific psychopathological conditions (e.g. externalising disorders, cluster B personality disorders and various types of antisocial behaviour).^1,9–11,44^ Individuals with antisocial personality disorder are also inclined to display interpersonal manipulation and low affectivity.^45^ In our study, we confirmed an association between these trait variables, although correlation coefficients were of limited size.

In our study, impulsivity, aggression and hostility rated on the BIS-11,^33^ BGLHA^31^ and BDHI^32^ also correlated negatively with adulthood: as age increased, there was a decrease in the ratings on these instruments. Several studies^46,47^ have shown that violent behaviour correlates positively with high levels of impulsivity and anger, variables that peak in adolescence and decrease with advancing age.^10,14,15^

Anger, evaluated using the STAXI-2,^35^ also decreased significantly with increasing age, confirming that younger age is associated with a higher level of anger. As some authors have shown,^47–51^ at least in people with psychotic disorders, anger might be the fundamental mediator between psychotic symptoms and the trigger of violent behaviour. Higher levels of anger, as detected with the STAXI-2, in young people and in people with personality disorders might be an extremely important therapeutic target: a reduction in anger might result in a reduction in the risk of aggressive and violent behaviour.

### Aggressive and violent behaviour

We evaluated the frequency and severity of aggressive and violent behaviour with the MOAS.^36^ Using a specific analytical methodology which made it possible to control the large number of MOAS ratings equal to 0, a linear increase in MOAS ratings was observed, especially in participants with a history of violence, who exhibited more aggressive and violent behaviour than those with no history of violence.^52^

Then, to understand how age was associated with violent behaviour, the sample was divided into three age groups: 18–29, 30–49 and ≥50 years of age (Fig. 2). Within these three groups, there was a marked difference: among participants belonging to the first group (18–29 years), only 2 out of 28 showed an average MOAS score equal to zero, i.e. an absence of violence. On the other hand, one-third of the second group (30–49 year) and half of the fourth group (≥50 years) showed a total absence of violent and aggressive behaviour over 1 year, i.e. with increasing age, there is a greater likelihood of low levels of aggression and violence. These results were confirmed even controlling for the gender effect.

### Clinical implications

These findings may have interesting clinical implications. Literature suggests that previous violent episodes are strongly associated with the risk of repeated violent episodes.^53^ Thus, recognising anger and its determinants in young patients might be an important step for the development of preventive interventions aimed at the reduction of the risk of future violent behaviour.^12,54–56^ Furthermore, the abundant literature on age at onset of mental disorders shows that most disorders have their onset in youth.^57^ Since the occurrence of violent behaviour can easily have serious legal, interpersonal, occupational and educational consequences, which may have long-lasting effects, the early and accurate recognition of the risk of violence should be a priority for mental health services. Specific psychotherapeutic interventions targeting anger and impulsivity may result in a lower risk of aggressive and violent acts in the future,^57^ ultimately improving the life of young patients. These clinical dimensions should also be taken into account for the prevention and treatment of SUDs, another modifiable risk factor closely associated with aggressiv and impulsivity.^17,58^

### Limitations

This study has a number of limitations. First, the duration of the observation period (1 year) may have reduced the possibility of detecting new aggressive and violent episodes and hence of identifying long-term predictors of such behaviour. Second, the MOAS assessment was based on the reports of patients’ treating clinicians or family members and not based on a direct 24 h observation. Thus, our results might have underestimated the occurrence of aggressive and violent behaviour in particular among out-patients, because the MOAS was not used to evaluate each individual aggressive episode. In any event, the restricted period of observation for each MOAS rating (2 weeks) makes it unlikely that relevant episodes of aggression or violence remained undetected, and the frequency of the MOAS ratings was the highest recorded so far in prospective cohort studies.^43^ Finally, this study evaluated a psychiatric population, not a sample from the general population; therefore the findings might not apply to the general population.

## Data Availability

The data that support the findings of this study are available from the corresponding author on request.

## Supplementary material

For supplementary material accompanying this paper visit http://dx.doi.org/10.1192/bjo.2021.1047. click here to view supplementary material
