# Supplementary material for: Young age and the risk of violent behaviour in people with severe mental disorders: prospective, multicentre study
Source: BJPsych Open. 2021 Dec 1;8(1):e1. doi: 10.1192/bjo.2021.1047 (PMC8693905; doi:10.1192/bjo.2021.1047)
Supplement: Supplementary file 1 [file S2056472421010474sup001.docx]

Supplementary Figure 1

Relationship between age and the area of the cumulative MOAS scores evaluated employing four different smoothing techniques.

|  |  |
| --- | --- |
|  |  |

Supplementary Figure 2

Cumulative means of Total MOAS scores (cMOAS) in three age groups over time separately for females and males.

| 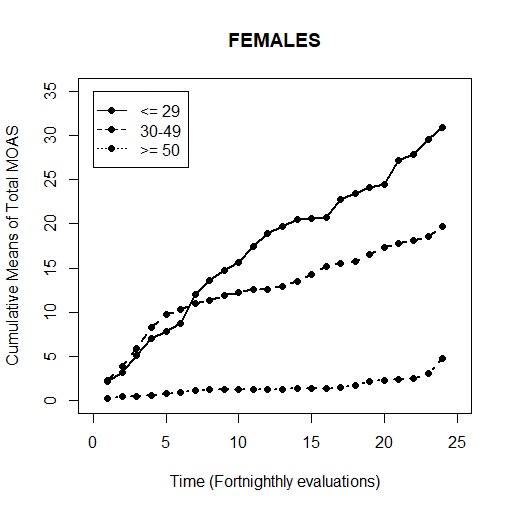 | 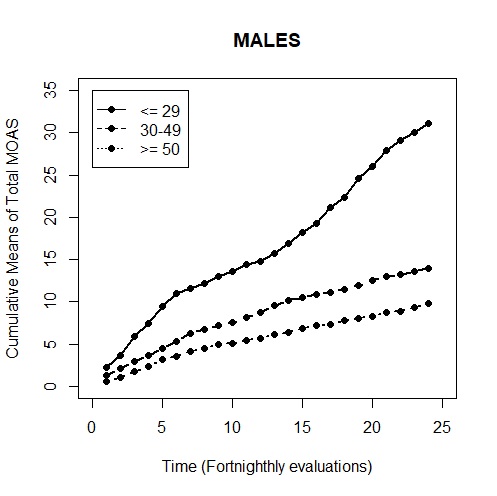 |
| --- | --- |

Supplementary Figure 3

Cumulative means of total MOAS by age categories and diagnostic groups.

|  |  |
| --- | --- |
|  |  |

Supplementary Figure 4

Cumulative means of total MOAS by age categories and different subgroups (history of violence and treatment setting).

|  |  |
| --- | --- |
|  |  |

Supplementary Figure 5

Cumulative means of total MOAS by age categories and different subgroups (diagnosis of substance user disorder and treatment with antipsychotic drugs).

|  |  |
| --- | --- |
|  |  |

Online Resource 1

Age according to patterns of medication prescription

| 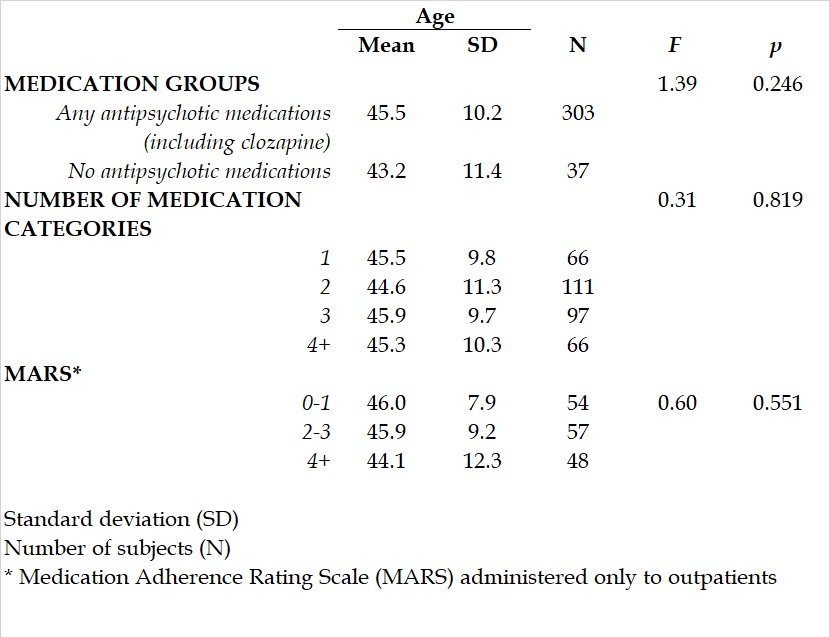 |
| --- |
